# Supplementary material for: Profiling Walnut Fungal Pathobiome Associated with Walnut Dieback Using Community-Targeted DNA Metabarcoding
Source: Plants (Basel). 2023 Jun 20;12(12):2383. doi: 10.3390/plants12122383 (PMC10305676; doi:10.3390/plants12122383)
Supplement: Supplementary file 1 [file plants-12-02383-s001.zip › plants-2405582-Supplementary File.pdf]

Supplementary Table S1. Total number of sequence reads obtained for each primer set and each mock replicate (-1, -2, -3) after read pre-processing step with and without the ITS2 region extraction additional step.

Primer set	Mock1			Mock2			Mock3			Mock4			Mock5		
	**M1-1**	**M1-2**	**M1-3**	**M2-1**	**M2-2**	**M2-3**	**M3-1**	**M3-2**	**M3-3**	**M4-1**	**M4-2**	**M4-3**	**M5-1**	**M5-2**	**M5-3**
ITS	35601	30814	27198	26126	27820	27587	40788	36961	32021	39048	39513	30496	42444	37605	36061
ITS1	38898	35619	36616	37986	37910	34191	50891	41730	48657	50890	52575	43680	60083	51699	37401
GTAA	49065	38493	41646	48554	55057	52562	42014	47906	39697	45967	40318	41336	43261	47527	41009
GTAAm	25062	24707	24207	41340	36624	40907	34170	35971	32265	30477	30591	29960	32809	34190	31484
Kyo	77209	141712	66163	63558	76730	78118	69227	77797	79702	75000	75652	74618	80320	73034	73046

Supplementary Table S2. Amplification conditions for metabarcoding sequencing. Conditions are common to all primer sets. Final volume of reaction was 25 µL. Conditions were determined by Génome Québec Innovation Center.

Master Mix Components	Final concentration	Amplification cycles
Qiagen 10X Buffer with 15 mM $MgCl_2$	1 X	Denaturing<br>96 cycles - 15 min<br><br>Annealing<br>96 cycles - 30 sec<br>52 cycles - 30 sec<br>72 cycles - 60 sec<br><br>Extension<br>72 cycles - 10 min
Roche DMSO	5 %	
dNTP mix 10 mM_NEB	0.2 mM	
Qiagen HotStarTaq 5U/µL	0.01 U/µL	
H_2O	q.s. 25 µL	
Forward primer	0.6 µM	
Reverse primer	0.6 µM	
eDNA	8 pM	

Supplementary Table S3. Optimal trimming parameters of mock and environmental samples indicated by FIGARO for each primer pair.

Primer set	Samples	Fwd reads trimming parameter (bp)	Rev reads trimming parameter (bp)	Maximum expected error (Fwd;Rev)	Read retention (%)	Score
ITS	Mock	292	220	4;4	78,62	60,02
ITS1	Mock	284	228	2;2	82,71	80,71
GTAA	Mock	281	229	3;4	82,78	69,78
GTAAm	Mock	282	228	3;4	82,21	69,20
	Environmental	280	230	3;4	82,8	69,80
Kyo	Mock	287	223	3;3	83,4	75,40
	Environmental	278	232	2;3	80,1	75,10

Fwd: Forward; Rev: Reverse.
Maximum expected error corresponds, for a given read, to the mean maximum number of errors that would be observed in a large collection of reads, where the error rate at each position is given by its quality score and occurs independently [142]. Score corresponds to a value assigned at the trimming parameters taking into account read retention values from which maximum expected errors values are subtracted [143].

Supplementary Table S4. Number (nb) of sequences for each species composing our local ITS2 database. The total number of sequences corresponds to the sequences retrieved from the GenBank database. Number of GTAAm and Kyo amplicons are the *in silico* amplicons obtained with the GTAA182fm/526r and ITS3/ITS4_KYO1 primer pairs. A total of 55 species and 71 species were represented in Kyo and GTAAm final databases respectively.

Classification	Species	Total nb of retrieved sequences	Kyo primers			GTAAm primers		
			Nb of amplicons	Nb of unique amplicons	Nb of retained amplicons for trees[c]	Nb of amplicons	Nb of unique amplicons	Nb of retained amplicons for trees[c]
Bionectriaceae	*Geosmithia flava*	4	0	0	0	2[b]	1	1
	Geo. lavendula	7	2	1	1	3[b]	1	1
	Geo. morbida	3	2	1	1	3[b]	1	1
Botryosphaeriaceae	*Botryosphaeria dothidea*	82	13	1	1	48	1	1
	Diplodia gallae	8	0	0	0	2	1	1
	D. mutila	33	12	5	5	22	5	2
	D. seriata	74	22	2	2	43	4	3
	Dothiorella iberica	47	15	2	2	33	6	2
	Dot. omnivora	3	0	0	0	0	0	0
	Dot. plurivora	5	1	1	1	2	2	2
	Dot. sarmentorum	36	7	2	2	7	2	2
	Dot. viticola	22	7	2	2	9	2	2
	Lasiodiplodia citricola	5	0	0	0	1	1	1
	L. mahajangana	6	0	0	0	4	2	2
	L. pseudotheobromae	35	2	2	2	26	2	2
	L. theobromae	96	51	2	2	76	4	4
	Neofusicoccum hellenicum	5	0	0	0	5	1	1
	N. luteum	47	3	2	1	38	3	2
	N. mediterraneum	50	2	2	2	27	5	3
	N. nonquaesitum	29	1	1	1	28	2	2
	N. parvum	95	21	3	2	60	6	4
	N. vitifusiforme	81	3	2	1	44	6	2
	Neoscytalidium dimidiatum	54	4	2	2	16	4	2

Supplementary Table S4. Continued.

Classification	Species	Total nb of retrieved sequences	Kyo primers			GTAAm primers		
			Nb of amplicons	Nb of unique amplicons	Nb of retained amplicons for trees[c]	Nb of amplicons	Nb of unique amplicons	Nb of retained amplicons for trees[c]
Diaporthaceae	*Diaporthe amygdali*	210	5	3	3	16	4	4
	Dia. australafricana	7	1	1	1	7	4	2
	Dia. biguttulata	2	0	0	0	0	0	0
	Dia. capsici	5	5	1	1	5	1	1
	Dia. cynaroidis	5	1	1	1	3	1	1
	Dia. eres	185	5	3	2	143	10	4
	Dia. foeniculina	124	28	5	5	109	8	4
	Dia. juglandicola	4	0	0	0	0	0	0
	Dia. novem	52	11	4	2	33	7	4
	Dia. rudis	79	20	3	1	68	7	2
	Dia. shennongjiaensis	6	2	1	1	2	1	1
Didymellaceae	*Epicoccum nigrum*[a]	52	8	3	3	46	4	2
Glomerellaceae	*Colletotrichum acutatum*	180	16	6	4	133	13	3
	C. fioriniae	84	3	3	3	80	2	2
	C. fructicola	92	32	2	1	85	3	2
	C. gloeosporioides	126	6	2	1	74	8	3
	C. godetiae	73	10	1	1	71	4	4
	C. kahawae	7	0	0	0	7	2	2
	C. nymphaeae	143	26	1	1	143	8	4
	C. siamense	106	8	3	2	94	6	3
	C. viniferum	33	8	3	3	19	7	3
Gnomoniaceae	*O. leptostyla*	9	0	0	0	7	3	2
Juglanconidaceae	*Juglanconis appendiculata*	7	7	2	2	7	2	2
	J. juglandina	7	6	1	1	6	1	1

Supplementary Table S4. Continued.

Classification	Species	Total nb of retrieved sequences	Kyo primers			GTAAm primers		
			Nb of amplicons	Nb of unique amplicons	Nb of retained amplicons for trees[c]	Nb of amplicons	Nb of unique amplicons	Nb of retained amplicons for trees[c]
Nectriaceae	*Fusarium avenaceum*	26	11	3	3	20	4	2
	F. chlamydosporum	11	2	2	1	6	4	1
	F. culmorum	18	5	4	3	11	4	2
	F. equiseti	22	9	2	2	21	3	2
	F. graminearum	232	21	2	2	227	12	2
	F. incarnatum	23	4	2	2	19	2	2
	F. juglandicola	6	4	1	1	6	1	1
	F. oxysporum	129	14	4	4	101	9	3
	F. proliferatum	70	28	3	2	53	10	2
	F. solani	212	26	4	4	59[a]	16	3
Plectosphaerellaceae	*Gibellulopsis nigrescens*[a]	33	1	1	1	6	2	2
Pleosporaceae	*Alternaria alternata*	138	5	3	3	132	7	4
	A. arborescens	20	3	2	2	18	3	3
	A. tenuissima	16	8	3	3	14	4	2
Togniniaceae	*Phaeoacremonium cinereum*	3	0	0	0	1	1	1
	P. fraxinopennsylvanicum	12	0	0	0	5	2	2
	P. italicum	0	0	0	0	0	0	0
	P. minimum	55	2	1	1	37	11	2
	P. parasiticum	15	4	3	3	11	3	2
	P. sicilianum	3	0	0	0	1	1	1
	P. tuscanum	2	0	0	0	2	1	1
	P. viticola	10	1	1	0	7	2	1

Supplementary Table S4. Continued.

Classification	Species	Total nb of retrieved sequences	Kyo primers			GTAAm primers		
			Nb of amplicons	Nb of unique amplicons	Nb of retained amplicons for trees[c]	Nb of amplicons	Nb of unique amplicons	Nb of retained amplicons for trees[c]
Valsaceae	*Cytospora atrocirrhata*	2	0	0	0	2	1	1
	Cyt. californica	29	0	0	0	0	0	0
	Cyt. ceratosperma	18	3	1	1	15	5	2
	Cyt. chrysosperma	23	1	1	1	11	1	1
	Cyt. cincta	3	0	0	0	3	3	3
	Cyt. gigalocus	2	0	0	0	2	1	1
	Cyt. joaquinensis	3	0	0	0	9	6	3
	Cyt. nivea	18	0	0	0	0	0	0
	Cyt. plurivora	17	0	0	0	0	0	0

[a] *E. nigrum* and *G. nigrescens* were the unique representative species of each genus ever isolated from environmental samples, so no other species of these genera were added to the databases for phylogenetic construction.

[b] Amplicons obtained with 2 mismatches allowed anywhere on primer sequences. [c] To improve clarity of trees, only some of the sequences were randomly kept.

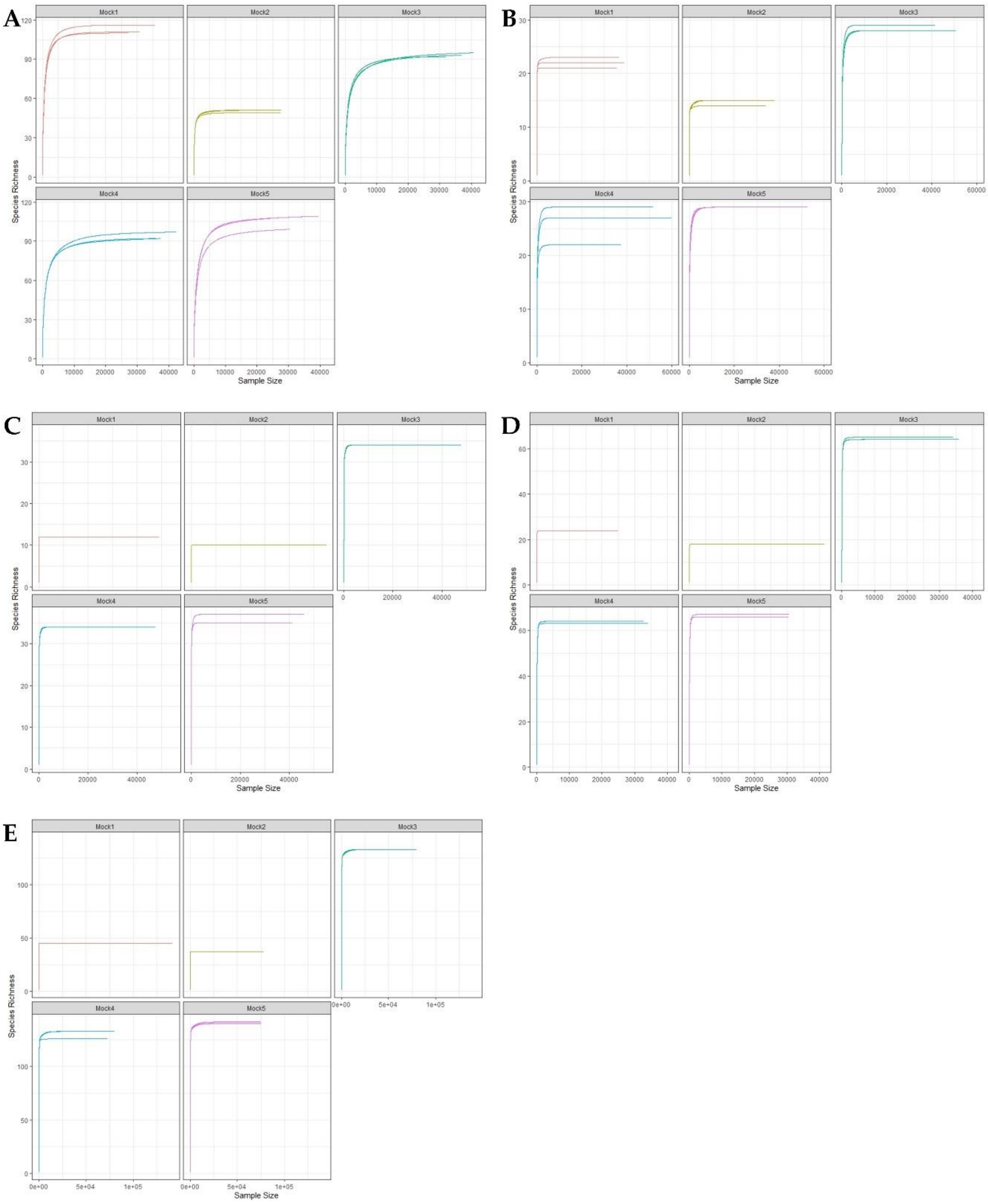

Supplementary Figure S1. Rarefaction curves of high-quality sequence reads for each mock community replicate. A: ITS set. B: ITS1 set. C: GTAA set. D: GTAAm set. E: Kyo set.
